# Gender Disparities in Receipt of HIV Testing Results in Six Sub-Saharan African Countries

**DOI:** 10.1089/heq.2018.0060

**Published:** 2018-12-26

**Authors:** Mulugeta Gebregziabher, Lin Dai, Caroline Vrana-Diaz, Abeba Teklehaimanot, Michael Sweat

**Affiliations:** ^1^ Department of Public Health Sciences, Medical University of South Carolina, Charleston, South Carolina.; ^2^ Department of Psychiatry and Behavioral Sciences, Medical University of South Carolina, Charleston, South Carolina.

**Keywords:** complex survey, HIV knowledge, HIV stigma, receipt of HIV test results, sub-Saharan Africa, weighted logistic

## Abstract

**Purpose:** Receipt of HIV testing results is vital for individuals to know their status and make decisions that would improve their access to HIV prevention, treatment, and care. The objective of this study is to determine the association of HIV testing and receipt of results with three key exposure variables (HIV stigma, HIV knowledge, and media use) stratified by gender and country.

**Methods:** Data from a random sample of adults aged 15–49 years from Burundi, Ethiopia, Kenya, Rwanda, Tanzania, and Uganda were abstracted from country-specific Demographic and Health Surveys or AIDS Indicators Surveys. Individuals were asked questions regarding demographics, socioeconomic status, sexual behaviors/attitudes, HIV knowledge, HIV stigma, and media-consumption. Weighted logistic regression was used to determine the association between receipt of HIV testing with key risk factors stratified by gender and country.

**Results:** Gender disparities in HIV testing and receipt of results, HIV stigma, and HIV knowledge remain high. More women have recently tested for HIV and received their results than men. HIV stigma was associated with decreased recent HIV testing in all six countries for women, and for men except in Ethiopia, Tanzania, and Uganda. HIV knowledge was positively related to recent testing in all countries, except Uganda for women and Kenya and Tanzania for men. In Burundi and Rwanda, women had more HIV knowledge than men, while in Kenya, Ethiopia, Tanzania, and Uganda, men had more HIV knowledge than women.

**Conclusion:** Given the importance of HIV testing for effective management of HIV in sub-Saharan Africa, it is crucial for these countries to exchange information on gender-specific policies and strategies that have the most impact on increasing HIV knowledge and decreasing HIV stigma.

## Introduction

HIV counseling and testing has been shown to be associated with reductions in transmission/acquisition behaviors for both HIV-infected and uninfected individuals,^1^ but it is only successful if individuals make informed decisions after receiving their results. Uptake of HIV testing remains relatively low in some countries in sub-Saharan Africa (SSA), with 40–80% ever tested and 20–53% testing and receiving results in the last 12 months.^2^ The World Health Organization (WHO) found between 2010 and 2014, only 48% of adults received HIV testing services in Africa, even though 52% of the global total of people living with HIV are found in SSA.^3,4^ This low testing uptake also affects linkage to care. By the end of 2016, only 60% of people living with HIV in SSA were accessing antiretroviral therapy.^5^ There is also increased risk of HIV infection by gender. As of 2015, young women accounted for 25% of new HIV infections among adults, and women accounted for 56% of new infections among adults in SSA.^3^ In response to the low testing rates, the WHO and the United Nations Program on HIV/AIDS issued guidance on provider-initiated HIV testing and counseling to support improved access to HIV prevention, treatment, and care in 2007,^6^ and in 2015 WHO issued a recommendation that trained lay providers can conduct rapid diagnostic HIV testing.^7^ However, there are limited studies that characterize the risk factors associated with HIV testing, and variations by region and gender.

Much of the knowledge about HIV testing uptake is drawn from small- to medium-sized surveys of risk groups or randomly selected populations. Since 1988, the Monitoring and Evaluation to Assess and Use Results Demographic and Health Surveys (MEASURE-DHS)^2^ project has served to fill the gap in large-scale data that allows for comprehensive analysis of risk factors associated with variations in HIV testing in over 60 countries. DHS collects information on HIV prevention knowledge, attitudes toward those living with AIDS, beliefs about AIDS transmission, and experiences with higher-risk sex. The large size of these datasets allows for within-country comparisons, the standardization of the surveys allows for between-country comparisons, and they provide the opportunity to identify unique associations with HIV testing outside the usual risk-based clusters.

Social stigma and discrimination associated with HIV is a major barrier to testing. Stigma not only deters those who are positive from seeking counseling, but also deters those at risk of infection from testing.^8–11^ HIV/AIDS stigma varies by country, community, religious groups, political climate, age, gender, and socioeconomic factors.^12^ However, there are no comprehensive studies that investigate the risk factors associated with HIV testing and its regional- and gender-specific variations in SSA.

The objectives of this study are therefore to determine the association of HIV testing and receipt of results with three key exposure variables (HIV stigma, HIV knowledge, and media use) stratified by gender and country of residence.

## Methods

Data were analyzed from six nationally representative surveys conducted in SSA between 2008 and 2011. The DHS was administered in Burundi, Ethiopia, Kenya, and Rwanda. The AIDS Indicator Survey (AIS) was administered in Tanzania and Uganda. Similar questions were included in both surveys. Participant sampling was made using a two-stage cluster randomized design, and is representative at the national, residence, and regional levels. Enumeration areas (EA) are drawn from census files with a sample of households drawn from each selected EA. All eligible household members are then surveyed. Individual-level survey weights were used during analysis to correct for the unequal probability of selection, and for dependence of observations within clusters due to the multistage cluster sampling strategy.

The 2010 Burundi DHS resulted in a sample of 8596 households (9389 women aged 15–49 years, 4280 men aged 15–59 years). The 2011 Ethiopia DHS consisted of 16,702 households (16,515 women aged 15–49 years, 14,110 men aged 15–59 years). The 2008/2009 Kenya DHS consisted of 9057 households (8444 women aged 15–49 years, 3465 men aged 15–59 years). The 2010 Rwanda DHS consisted of 12,540 households (13,671 women aged 15–49 years, 3329 men aged 15–59 years). The 2010 Tanzania AIS consisted of 10,040 households (10,967 women aged 15–49 years, 8352 men aged 15–49 years). The 2011 Uganda AIS consisted of 11,340 households (12,153 women aged 15–49 years, 9588 men aged 15–59 years). We restricted samples to men aged 15–49 years to equally estimate the associations of factors by gender.

Data were obtained from the official DHS repository (www.dhsprogram.com/data). For the DHS, individual recode files for men and women are appended, and merged with results of the HIV testing module.^13^ The AIS dataset includes records for men and women and HIV testing-related variables. The analysis dataset included appended individual files from each country. To check for accuracy, all outcomes were compared to published reports by DHS, or their online tools were used for generating queries. In one case we observed an unlikely level of missing response to a key outcome for the DHS in Burundi. The authors contacted DHS, and were informed of a coding error reflected in the provided dataset. DHS supplied the code required to correct the item response. Institutional Review Board approval was waived due to the publicly available nature of the data.

### Outcome

The outcome variable is receipt of HIV test (based on response to “Have you tested for HIV/AIDS in the last 12 months and received the results of the test?”).

### Covariates

Three key predictors were included (HIV stigma, HIV knowledge, and media use), and demographic, socioeconomic, and behavioral variables were included as covariates. These included age, gender, education (none, primary, secondary, and higher), wealth (dichotomized at median wealth), place of residence (urban, rural), marital status (currently married, separated, and never married), and sexually transmitted infection (STI) burden (self-report score of any STI/genital ulcer/genital discharge in the last 12 months and number of partners [none, 1, 2+]). HIV stigma is at least one negative response to one of four stigma variables (stigma 1=if a member of your family became sick with HIV/AIDS would you be willing to care for him in your household?; stigma 2=if you knew that a shopkeeper or food seller had AIDS, would you buy fresh vegetables from him or her?; stigma 3=if a female teacher has the AIDS virus but is not sick, should she be allowed to continue teaching in school?; stigma 4=if a member of your family became infected with AIDS, would you want it to remain a secret?). HIV comprehensive knowledge is a composite score based on correct responses to five questions^14^ that include two ways to prevent AIDS (using condoms and having sex only with one faithful uninfected partner) and three questions accepting correct HIV beliefs (healthy-looking person can have the AIDS virus, AIDS cannot be transmitted by mosquito bites, AIDS cannot be transmitted by supernatural means). Media use is an indicator of using at least one of these media (radio, TV, and newspapers/magazines) on a weekly basis or at any time.

### Statistical analysis

Preliminary analyses were conducted to describe country characteristics. Descriptive statistics were calculated for HIV stigma, knowledge, and media use by country, gender, and recent HIV testing and receiving results.

**FIG. 1.**
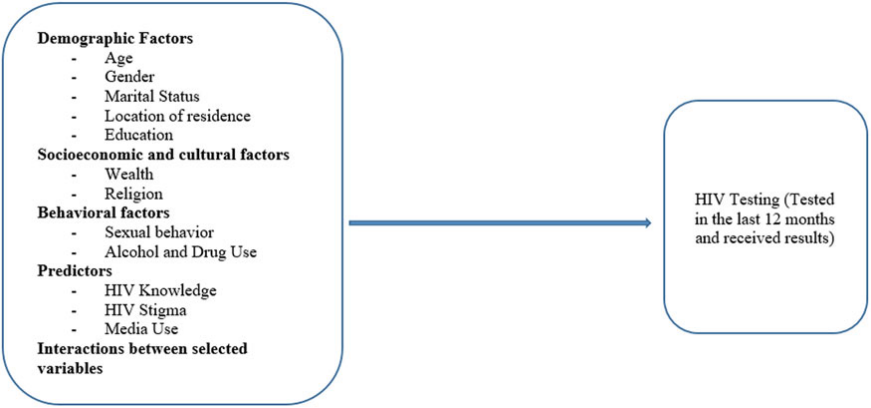
Conceptual model between demographic variables, HIV knowledge, HIV beliefs, HIV stigma, and HIV testing and receipt of results.

To estimate the association between key predictors and the outcome, weighted logistic regression that properly accounts for survey design was used. The modeling framework followed the conceptual model depicted in Figure 1. In each country, for individual *j* in cluster *i*, ${y_{ij}}$ denotes the observed response variable, ${x_{ij}}$ denotes the vector of explanatory variables, $\beta$ denotes vector of parameters to be estimated, ${w_{ij}}$ denotes the sampling weight, and ${ \pi _{ij}}$ denotes the expected probability of receipt of HIV testing results, then the logistic model can be written as follows:

\begin{align*}
\log \left( { { \frac { { \pi _ { ij } } }  { 1 - { \pi _ { ij } } } } } \right) = \beta { x_ { ij } } 
\end{align*}

The parameters in the above model were estimated using pseudo-log likelihood^16^ as shown:

\begin{align*}
l ( \theta ) = \sum \limits_i^{} { \sum \limits_j^{} {{w_{ij}}} } ( ( \log ( { \pi _{ij}} ) ) {y_{ij}} + ( \log ( 1 - { \pi _{ij}} ) ) ( 1 - {y_{ij}} ) )
\end{align*}

Estimates were weighted by sampling weights (${w_{ij}}$; provided as part of the datasets) to account for the nature of the survey data (made using the weight statement of PROC GENMOD in SAS 9.4).^16^

Following the conceptual framework depicted in Figure 1, modeling was derived via a sequential approach starting from unadjusted analysis and then including demographic, socioeconomic status, behavioral covariates, and the primary predictors. Within each of the three models (one for each primary predictor), optimal combinations of covariates, including interaction effects (including region), were identified and used for further analysis based upon potential confounders, model fit, or increased standard error of the primary predictors' parameter estimates. The association estimates were calculated as odds ratio (OR) with 95% confidence interval (CI). Each model was assessed for collinearity and goodness-of-fit using residual analysis. Archer et al.^15^ and Hosmer and Lemeshow^17^ goodness-of-fit test was used. The more closely predicted frequency and observed frequency match, the better the fit.

## Results

Overall, the final sample size for this analysis was 42,704 men aged 15–49 years, and 70,134 women aged 15–49 years. Table 1 shows the population characteristic of each country by gender. The majority of people are younger (age <30 years), over 50% are married, over 70% live in rural areas, and over 60% are above median wealth. HIV stigma, HIV knowledge, and HIV behavior are different by country and gender. Overall, Rwanda had the lowest percent of women with at least one form of stigma (45.4%) while Ethiopia (76.4%) and Uganda (76.1%) had the highest. The figures were similar for men. However, in all countries, men had lower percentages of a negative response to at least one of the four stigma questions than women. In Kenya and Rwanda, women had higher levels of correct beliefs and knowledge about HIV compared with other countries. The lowest levels of HIV knowledge were observed in Ethiopian women and highest in Rwanda. These figures are similar in men, with Kenya, Rwanda, and Tanzania showing the highest levels and Ethiopia showing the lowest levels. Except in Ethiopia, women had higher or relatively equal comprehensive HIV knowledge compared with men (41.4% in Ethiopian men vs. 30.2% for women). STI burden was the highest in Uganda and Rwanda in both men and women, but women have a higher STI burden in all but one country compared with men. However, the rate of media use was the highest in Rwanda and Tanzania and the lowest in Ethiopia, with men showing higher rates of media use than women.

**Table 1.** Sociodemographic Characteristics (Percent of People in Each Category) for Each Country Stratified by Gender

	Burundi	Ethiopia	Kenya	Rwanda	Tanzania	Uganda	*p*	Burundi	Ethiopia	Kenya	Rwanda	Tanzania	Uganda	*p*
Country	Women							Men						
Age														
15–19 years	25.37	23.23	20.89	21.71	22.72	21.99	<0.001	24.43	22.01	23.31	25.22	25.37	23.96	<0.001
20–24 years	19.78	18.29	20.65	19.7	17.63	19.41		20.52	18.11	19.06	20.35	17.96	15.97	
25–29 years	16.94	19.28	16.89	18.26	16.55	17.23		15.41	17.67	14.99	18.37	13.71	15.31	
30–34 years	11.5	12.71	14.03	13.32	12.96	13.13		12.37	13.07	14.84	12.75	12.11	13.35	
35–39 years	11.22	11.85	11.00	10.53	12.92	12.21		10.14	12.26	11.05	8.57	12.43	12.81	
40–44 years	7.85	7.96	8.61	8.43	9.52	8.48		9.07	9.40	8.96	7.62	10.44	10.17	
45–49 years	7.34	6.67	7.94	8.05	7.69	7.55		8.05	7.47	7.79	7.13	7.98	8.42	
Marital status														
Never married	35.06	26.74	30.13	39.28	26.74	23.5	<0.001	46.63	43.84	45.97	50.92	43.62	37.11	<0.001
Married	55.94	61.76	59.63	49.95	62.2	63.52		52.03	52.65	50.25	47.08	51.98	56.79	
Separated (divorced + widowed)	9.00	11.50	10.23	10.78	11.06	12.98		1.34	3.51	3.79	2.00	4.41	6.10	
Education attainment														
None	40.79	50.12	14.1	15.03	17.78	14.61	<0.001	25.03	28.44	5.17	10.18	8.63	5.37	<0.001
Primary	42.07	35.46	52.44	67.88	61.16	58.97		48.86	49.22	51.85	68.20	64.73	56.84	
Secondary+	17.14	14.42	33.46	17.10	21.06	26.42		26.11	22.34	42.98	21.62	26.63	37.80	
Population density														
Rural (vs. urban)	77.5	67.73	68.93	82.66	76.04	78.9	<0.001	69.36	69.58	68.5	81.00	77.23	79.97	<0.001
Wealth														
≥Median	63.59	62.99	65.15	62.22	65.82	62.54	<0.001	69.80	65.38	66.78	68.08	66.86	61.87	<0.001
HIV stigma														
Stigma 1	20.65	15.74	9.35	3	5.99	6.24	<0.001	10.19	7.23	4.87	2.5	3.12	4.51	<0.001
Stigma 2	26.76	59.15	32.76	16.06	37.68	26.06	<0.001	13.65	45.83	22.31	9.79	28.45	18.05	<0.001
Stigma 3	22.96	34.21	22.55	11.17	13.27	18.77	<0.001	14.26	26.17	19.65	10.53	15.69	18.70	<0.001
Stigma 4	15.24	41.28	45.00	32.71	56.41	65.5	<0.001	14.10	36.52	28.54	21.04	41.77	53.14	<0.001
Negative response to at least one of four stigma	50.86	76.39	64.31	45.44	71.82	76.14	<0.001	35.14	68.27	49.49	33.32	58.44	64.02	<0.001
HIV knowledge														
Correct beliefs	66.23	42.30	71.66	75.66	69.76	59.30	<0.001	65.40	54.27	75.64	73.65	74.18	61.29	<0.001
Comprehensive	58.91	30.20	59.59	64.14	56.98	53.75		57.91	41.38	63.74	56.80	58.92	56.28	
Media														
Any media	82.08	65.01	85.12	93.26	92.13	89.65	<0.001	94.00	84.47	97.38	98.03	95.68	95.16	<0.001
Sexual behavior														
STI burden	10.59	2.93	7.39	10.73	7.66	32.96	<0.001	2.44	2.79	2.28	10.85	5.12	15.30	<0.001
Number of partners														
0 Partners	40.37	37.05	28.93	44.67	24.81	25.22	<0.001	43.09	39.98	28.27	44.08	27.14	26.81	<0.001
1 Partner	59.37	62.63	69.84	54.76	71.85	71.89		53.58	55.65	61.30	51.93	53.84	55.21	
>1 Partner	0.26	0.33	1.23	0.57	3.34	2.89		3.33	4.38	10.43	3.99	19.02	17.99	
HIV testing outcomes														
Tested/received results within last 12 months	18.68	20.00	29.29	38.63	30.26	23.92	<0.001	10.99	19.92	22.33	36.62	26.54	23.17	<0.001

STI, sexually transmitted infection; stigma 1, if a member of your family became sick with HIV AIDS would you be willing to care for him in your household?; stigma 2, if you knew that a shopkeeper or food seller had AIDS, would you buy fresh vegetables from him or her?; stigma 3, if a female teacher has the AIDS virus but is not sick, should she be allowed to continue teaching in school?; stigma 4, if a member of your family became infected with AIDS, would you want it to remain a secret?

Table 1 also shows the variation in rates of recent HIV testing and receiving results. The percent of recent HIV testing and receiving results is the highest in Rwanda, followed by Tanzania, Uganda, Rwanda, Ethiopia, and then Burundi. In all countries, the rates of recent HIV testing and receiving results is higher for women compared to men.

Table 2 presents regional distributions of recent HIV testing and receiving results by demographic variables stratified by gender. The rates of HIV testing and receiving results peak at 20–24 years in women and 25–29 years in men in almost all countries, and are the lowest among 45–49 years old. Married people were more likely to have recently tested and received results compared with never married or separated people. Never married women had higher testing rates and receipt of results than never married men in all countries except for Ethiopia (22.9% vs. 23.1%, respectively). People with secondary or higher education were more likely to have recently tested and received results than those with lower education. In Rwanda, recent HIV testing and receipt in rural areas (38.5% for women, 37.7% for men) were close to the percentages in urban areas (37.9% for women, 36.9% for men), while in other countries there were disparities of HIV testing and receipt of results between rural and urban areas. Men and women with higher wealth were more likely to have tested for HIV and received results than those with lower wealth.

**Table 2.** Percent HIV Tested (Recently Tested and Received HIV Test Results in Past 12 Months) for Each Country, Stratified by Gender

	Burundi	Ethiopia	Kenya	Rwanda	Tanzania	Uganda	*p*	Burundi	Ethiopia	Kenya	Rwanda	Tanzania	Uganda	*p*
Country	Women							Men						
Age														
15–19 years	12.2	19.1	18.2	27.1	19.3	19.2	0.045	6.9	16.6	13.0	24.1	11.4	11.7	<0.001
20–24 years	26.3	28.5	39.7	47.0	37.6	28.8		18.1	28.2	27.6	41.0	27.3	26.4	
25–29 years	26.0	27.0	39.3	46.7	37.2	26.9		18.0	26.3	31.4	46.9	32.4	31.5	
30–34 years	25.5	22.3	36.2	42.4	34.3	24.2		16.3	22.7	28.2	43.5	32.2	29.2	
35–39 years	21.7	20.4	24.3	39.3	29.4	21.3		11.9	20.8	27.0	40.8	30.6	28.7	
40–44 years	14.0	16.7	24.4	34.4	22.7	24.0		12.4	20.1	20.6	38.5	29.9	23.2	
45–49 years	8.4	13.7	17.8	25.8	16.7	21.0		9.8	18.2	18.2	35.0	24.6	21.4	
Marital status														
Never married	13.4	22.9	21.8	31.1	20.3	21.8	0.179	12.3	23.1	20.9	30.4	17.5	17.2	<0.001
Married	23.9	21.7	33.8	44.7	32.2	24.0		14.4	21.0	26.3	44.8	31.2	27.8	
Separated (divorced + widowed)	19.1	24.6	30.8	36.3	31.1	26.7		5.9	28.5	16.3	47.4	27.4	23.1	
Education level														
No education	15.8	13.9	18.4	35.3	21.3	20.0	<0.001	8.5	11.7	9.4	38.3	17.2	13.2	<0.001
Primary	20.6	26.2	29.9	37.7	30.2	21.2		12.7	21.4	19.7	36.3	25.2	19.8	
Secondary or higher	27.1	42.4	35.1	43.9	31.3	31.8		19.1	37.4	29.7	41.1	27.1	30.8	
Residence														
Urban	30.1	38.5	36.6	37.9	35.8	32.2	<0.001	19.6	34.0	29.3	36.9	31.7	29.7	<0.001
Rural	16.8	14.6	26.9	38.5	26.7	21.6		10.5	17.0	20.7	37.7	23.1	22.0	
Wealth index														
<50%	14.7	11.1	25.3	37.1	24.7	20.2	<0.001	9.6	12.5	18.3	35.7	21.0	21.5	<0.001
≥50%	22.7	29.0	32.4	39.2	31.0	26.0		14.9	27.3	25.9	38.4	27.1	24.9	
Stigma 1														
No	21.5	25.8	31.5	38.9	30.0	24.8	0.008	13.8	23.6	24.2	37.9	25.7	24.7	0.465
Yes	13.6	10.6	19.6	24.8	16.8	16.6		9.6	11.0	15.0	22.5	10.1	16.0	
Stigma 2														
No	21.8	34.8	32.7	39.5	32.4	25.7	<0.001	14.1	28.5	25.0	38.7	27.4	25.9	0.187
Yes	14.6	15.6	25.0	32.9	23.7	20.3		8.5	15.7	18.4	26.6	19.8	17.0	
Stigma 3														
No	21.5	30.4	32.8	39.4	30.7	25.5	<0.001	13.9	26.6	25.9	38.4	26.1	26.1	<0.001
Yes	14.6	12.2	22.1	31.6	20.6	19.3		10.6	12.5	13.7	29.6	21.2	15.3	
Stigma 4														
No	19.5	25.6	31.3	38.9	29.4	26.3	<0.001	13.0	23.8	23.8	37.8	27.6	28.1	<0.001
Yes	21.5	20.4	28.7	37.4	29.0	23.1		14.3	20.2	22.6	36.5	21.9	20.6	
Negative response to at least one of four stigma														
No	22.4	37.9	34.2	40.1	32.8	27.6	<0.001	13.3	30.6	27.0	38.2	29.6	30.4	<0.001
Yes	17.3	18.8	28.0	36.4	27.6	22.7		11.6	17.7	19.2	33.1	22.0	20.4	
No incorrect beliefs about AIDS														
No	17.6	21.8	27.1	38.0	26.5	23.7	0.002	11.7	18.7	22.5	34.8	21.6	21.7	0.054
Yes	21.9	32.9	32.8	39.3	32.3	26.4		14.8	29.5	24.6	39.3	27.5	28.0	
Comprehensive correct knowledge about AIDS														
No	17.7	25.8	27.8	38.7	28.4	24.9	0.014	12.3	21.8	21.5	35.3	23.5	22.7	0.602
Yes	23.1	36.0	35.6	39.7	35.1	27.4		15.3	30.4	26.4	40.7	29.2	28.7	
STI in last 12 months if sexually active in last 12 months														
No	28.2	28.5	35.8	46.6	43.7	27.2	0.003	21.3	27.9	25.1	49.3	33.3	26.7	0.04
Yes	32.0	31.6	31.7	48.6	44.7	28.6		12.5	29.1	52.9	37.5	21.3	24.0	
Number of partners														
0	12.1	19.9	16.9	28.6	14.4	18.7	<0.001	10.6	20.7	13.7	27.8	11.9	13.9	0.003
1	24.9	23.6	35.0	46.2	33.5	25.5		15.0	23.3	26.6	44.3	30.0	26.5	
2+	41.7	42.6	46.1	57.7	35.9	27.3		21.3	21.7	31.3	56.4	29.9	29.1	
At least one media source with any use														
No	15.1	11.3	19.4	35.0	21.8	17.3	<0.001	8.7	8.9	11.4	31.3	16.6	19.2	<0.001
Yes	20.8	28.3	31.8	38.7	29.5	24.6		13.6	24.6	23.7	37.6	25.4	23.8	

Table 2 also shows recent HIV testing and receiving results by HIV stigma, HIV knowledge, media use, and other sexual behaviors. Overall, men or women with no stigma in all four stigma situations are more likely to have recently tested and received their results. Men and women who have correct beliefs or comprehensive knowledge about HIV/AIDS are more likely to have recently tested for HIV and received results. Men and women who had any media use are more likely to have recently tested and received their results than those who did not.

**FIG. 2.**
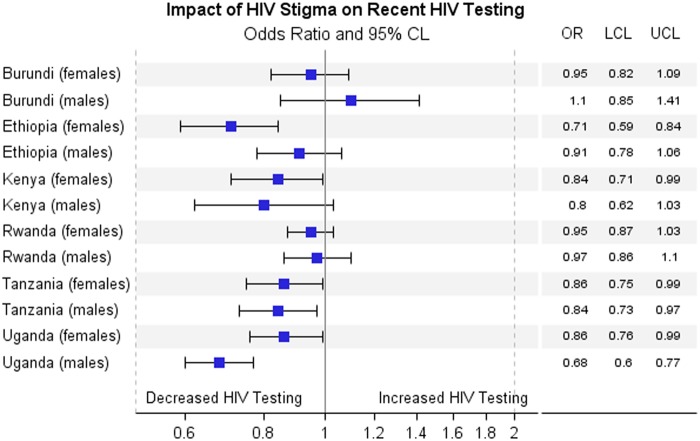
Adjusted association between impact of HIV stigma and recent testing with receipt of results. CL, confidence limit; LCL, lower confidence limit; OR, odds ratio; UCL, upper confidence limit.

**FIG. 3.**
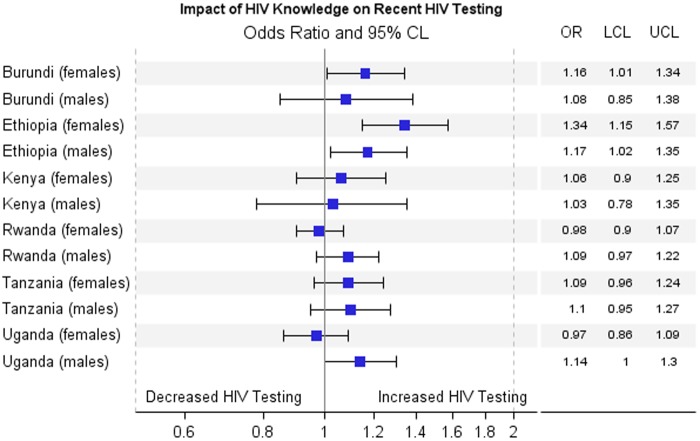
Adjusted association between impact of HIV knowledge and recent testing with receipt of results.

**FIG. 4.**
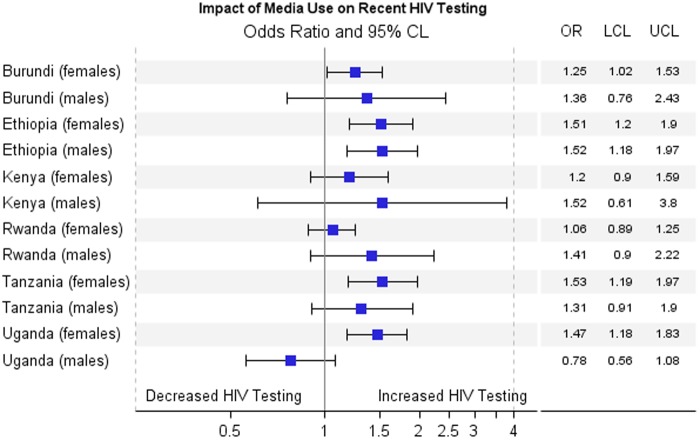
Adjusted association between impact of media use and recent testing with receipt of results.

Figures 2–4 show results for the adjusted association between the primary predictors and recent HIV testing with receipt of results. In Figure 2, HIV stigma score in men is negatively associated with recent HIV testing and receipt of results in Tanzania (OR=0.84; 0.73–0.97) and Uganda (OR=0.68; 0.60–0.77), but is not significant for men in Burundi, Ethiopia, Kenya, and Rwanda. HIV stigma score in women is negatively associated with testing and receipt of results in Ethiopia (OR=0.71; 0.59–0.84), Kenya (OR=0.84; 0.71–0.99), Tanzania (OR=0.86; 0.75–0.99), and Uganda (OR=0.86; 0.76–0.99), but not significant in Rwanda or Burundi. In Figure 3, HIV knowledge is positively associated with HIV testing and receipt of results in Ethiopian women, (OR=1.34; 1.15–1.57), Ethiopian men (OR=1.17; 1.02–1.35), Burundi women (OR=1.16; 1.01–1.34), and Ugandan men (OR=1.14; 1.00–1.30), showing that people with comprehensive HIV knowledge are more likely to have recently tested and received results. In Figure 4, media use is positively associated with HIV testing and result receipt within the past 12 months in Burundi (women), Ethiopia (men and women), Tanzania (women), and Uganda (women), indicating that people with more access to media are more likely to have recently tested and received results.

## Discussion

Our results show that there is significant regional and gender variation in rates of HIV testing and receipt of results in six SSA countries, as well as in the association between risk factors and HIV testing. The rates of recent HIV testing and received results is the highest in women compared with men in all countries.

HIV stigma was associated with a decreased likelihood of recent HIV testing and receipt of results in all six countries for women except Burundi, and for men in Tanzania and Uganda. This is consistent with other studies that showed people with a high degree of stigma toward HIV are less likely to test for fear of being stigmatized (from peers or health care providers).^8–11,18–20^ HIV knowledge was positively related to HIV testing and receiving results in Burundi and Ethiopia for women, and Ethiopia and Uganda for men. These results are consistent with other studies about the effect of HIV knowledge on HIV testing.^21,22^ In Ethiopia, media use was positively related to HIV testing and receiving results in both men and women, although media use was also positively related to HIV testing and receiving results in women in Burundi, Tanzania, and Uganda. This is supported by previous studies that showed media use is positively associated with HIV testing partly because media use is a common facilitating factor for testing, and for many people their major source of HIV information is mass media.^23–25^ The information provided by newspaper, radio, or television may bring changes in HIV knowledge and stigma, resulting in behavior change.^26,27^

Gender disparities in HIV testing and receiving results, HIV stigma, HIV knowledge, and STI burden remain high in most countries. In these six countries, more women than men have tested for HIV and received their results within the last 12 months, likely driven by antenatal HIV testing programs.^12,27–29^ This also shows the importance of not only testing for HIV, but also to receive the results. Men are more likely to have less HIV sigma than women in all six countries. For HIV knowledge, the gender disparities differ by country. In Burundi and Rwanda, women had more HIV knowledge than men, while in Ethiopia, Kenya, Tanzania, and Uganda, men had more HIV knowledge than women. This might be due to the HIV education or polices related to HIV in each country, and how education can target men and women differently. Women had a higher STI burden compared with men in all countries but Rwanda (where they are similar), suggesting that STI-related health care and education needs to pay more attention to women.

These results are incredibly important, as they show the necessity of reducing HIV stigma and increasing HIV comprehensive knowledge to increase both HIV testing and receipt of those HIV test results. Additional research is needed in these six countries to identify the most effective method of increasing HIV testing and receipt of results by targeting HIV beliefs, HIV knowledge, and HIV stigma.

A limitation of the study is the difference in survey timing. The data and reports for the six countries in this study were released between 2008 and 2011. This range could have an impact on the observed regional variation in rates of testing and receiving results, but this should not have a meaningful impact on the risk factors associated with HIV testing. Another limitation is the ambiguity in the HIV stigma definition. HIV stigma could refer to (1) perceived stigma in the community by the individual, (2) experienced stigma, or (3) personal stigma that the interviewee has toward persons with HIV. The interviewee's answer to the stigma questions could refer to any of these, so the interpretation needs to be understood in this light. Assessing trends over time with a more recent survey could improve the significance of the results.

Furthermore, the CIs of the parameter estimates for men are wider compared with women, especially in Burundi, Kenya, and Rwanda. This might be due to the disparities of sampling weight among genders. For Burundi, Kenya, and Rwanda, men are chosen from one-half to one-third of the selected households, while women are chosen from all selected households. The sampling weight for men in those countries is much larger compared with women, and the relatively larger sampling weight will bring relatively larger variance estimates, leading to wider CIs.

## Conclusion

The key driving factors for variation in recent HIV testing and receiving results are HIV stigma and HIV knowledge after covariate adjustment. It is prudent for researchers to investigate and understand the underlying factors that contribute to gender-specific differences in HIV stigma and knowledge. Given the importance of HIV testing for effective management of HIV in SSA, it is crucial for these countries to exchange information on what set of policies and strategies have the most impact on HIV knowledge and HIV stigma, giving special attention to those gender-specific targeted strategies.

## Abbreviations Used

AIS: AIDS Indicator Survey
CI: confidence interval
EA: enumeration areas
MEASURE-DHS: Monitoring and Evaluation to Assess and Use Results Demographic and Health Surveys
OR: odds ratio
SSA: sub-Saharan Africa
STI: sexually transmitted infection
WHO: World Health Organization
